# Supplementary material for: Common mechanism of thermodynamic and mechanical origin for ageing and crystallization of glasses
Source: Nat Commun. 2017 Jun 29;8:15954. doi: 10.1038/ncomms15954 (PMC5493766; doi:10.1038/ncomms15954)
Supplement: Supplementary Information [file ncomms15954-s2.pdf]

File name: Supplementary Information
Description: Supplementary Figures.

File name: Supplementary Movie 1
Description: Breakage process of force chains during avalanche

SUPPLEMENTARY FIGURES

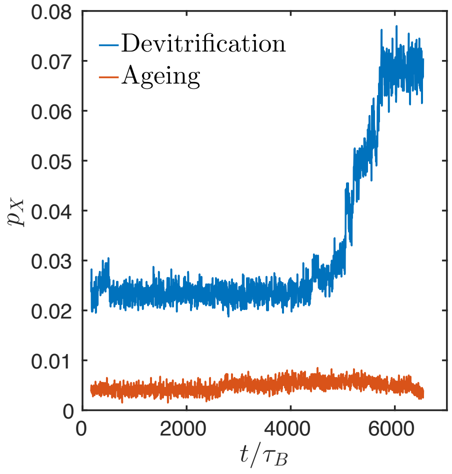

Supplementary Fig. 1. **Crystallinity over time.** The evolution of the proportion of crystalline particles p_X over time for a randomly selected trajectory for the monodisperse, devitrifying case and the polydisperse, ageing case. Note that the crystallinity undergoes intermittent growth during devitrification but not during ageing.

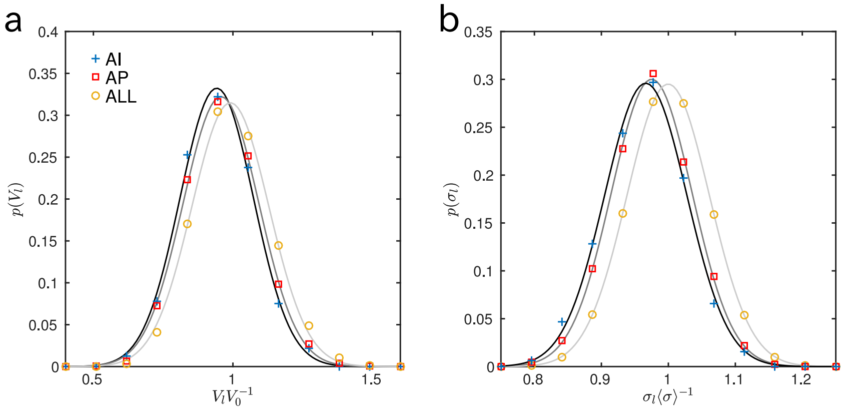

Supplementary Fig. 2. **Voronoi volume or size? a,** Distribution of local Voronoi volume for AIs, APs and all particles in ageing polydisperse trajectories, averaged over 50 events. **b,** Distribution of particle size for AIs, APs and all particles. Note that the resulting low local volume fraction for AIs and APs must be driven by the smaller size of particles, since the Voronoi volumes for AIs and APs are not clearly separated.

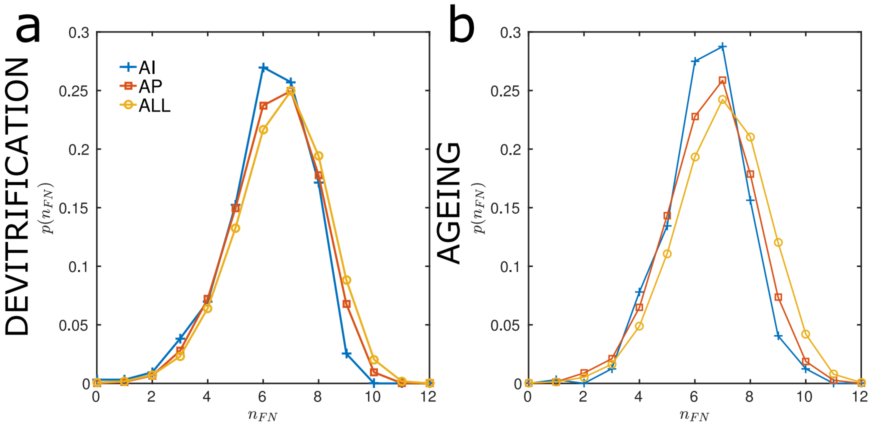

Supplementary Fig. 3. **Force neighbours.** Distribution of the number of force neighbours that particles have for AIs, APs and over all particles before avalanche events for the monodisperse (**a**) and polydisperse (**b**) cases. APs and AIs have a slightly smaller number of force neighbours in both cases, as expected from their lower Voronoi density.

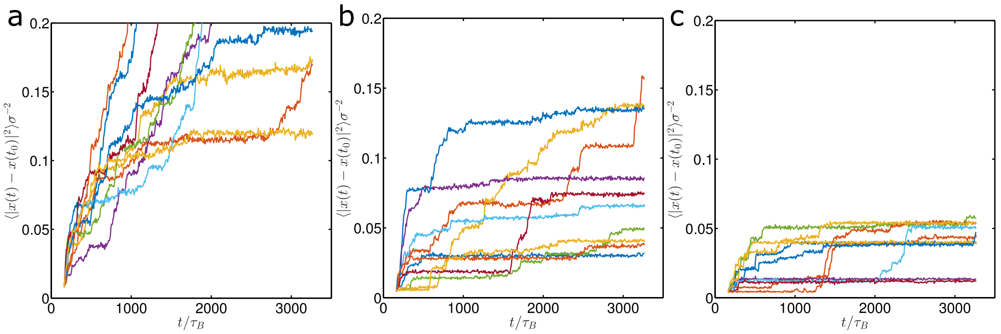

Supplementary Fig. 4. **Likelihood of avalanche events vs.** ϕ**.** The mean-squared displacement of particles over time at different volume fractions for 10 independent trajectories. **a,** 63%; **b,** 65%; **c,** 66%. Different colours indicate different trajectories. There is a clear reduction in mobility, as well as an increased likelihood for the trajectories to stay in a metastable (plateau) state for more of the time domain shown.

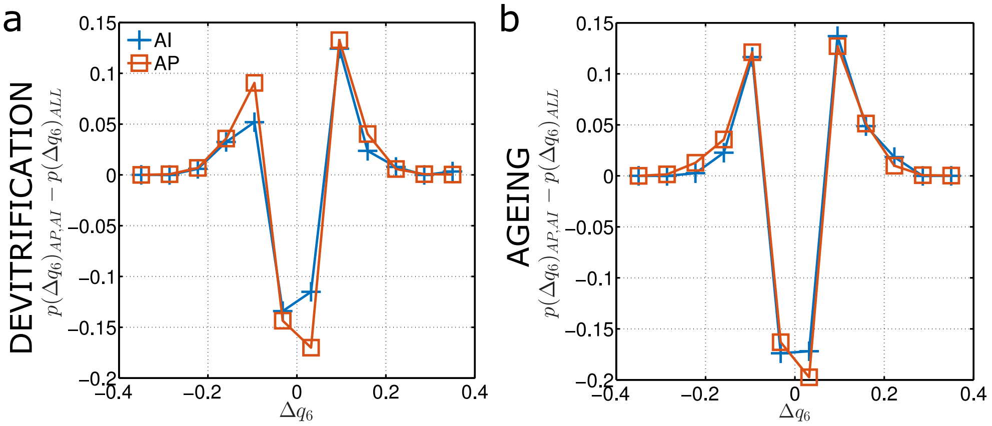

Supplementary Fig. 5. **Change in** q_6 **for AIs and APs vs. all particles. a, b,** The difference between the probability of Δq_6 for AI/AP particles and the probability of Δq_6 for the entire particle population for devitrification and ageing events, respectively. Though the distinction between AIs and APs is clear for the monodisperse system, this is not the case for the polydisperse system (see the text).

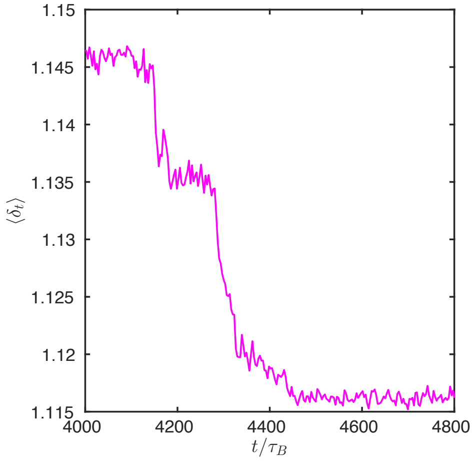

Supplementary Fig. 6. **Collective motion.** The average of the collective motion parameter δ over time, which decreases over the course of the avalanche. The event shown follows the same polydisperse trajectory as the one used for analysis of nearest neighbour and force neighbour changes in Fig.4a.

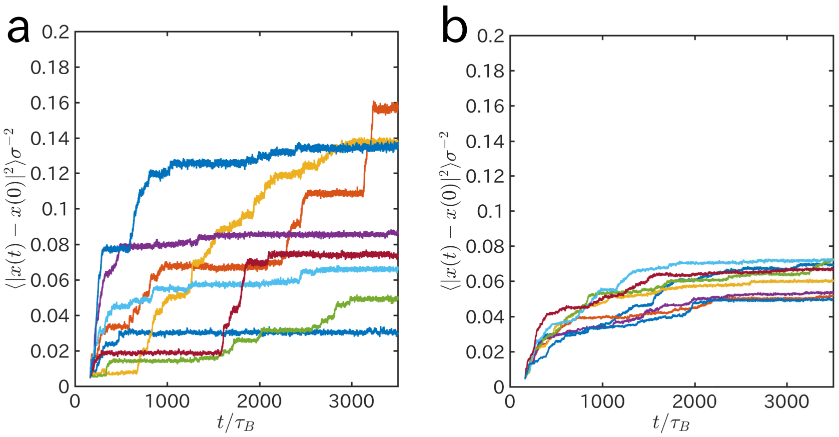

Supplementary Fig. 7. **Mean-squared displacements for** $n = 4000$ **and** $n = 16000$**.** MSDs found from eight independent trajectories for (**a**) $n = 4000$ and (**b**) $n = 16000$ system sizes. It can be seen that a larger system gives smaller events with less well-defined plateaux. This is due to events involving the same number of particles giving rise to smaller steps for larger n, and an increased chance of more events in a given time; events initiate while others have not relaxed yet.
